# Omalizumab for pediatric allergic asthma: evidence-based insights on efficacy and safety from a meta-analysis of randomized trials

**DOI:** 10.3389/fphar.2026.1795193

**Published:** 2026-09-24

**Authors:** Jinbiao Han, Zhuan Zou, Shanshan Wu, Bin Chen

**Affiliations:** 1 Department of Emergency, West China Second University Hospital, Sichuan University, Chengdu, China; 2 Key Laboratory of Birth Defects and Related Diseases of Women and Children, Sichuan University, Ministry of Education, Chengdu, China; 3 Department of Emergency, Meishan Maternity and Child Healthcare Hospital, Meishan, Sichuan, China

**Keywords:** adolescent, allergic asthma, meta-analysis, omalizumab, pediatric, randomized controlled trials

## Abstract

**Objectives:**

This meta-analysis aimed to synthesize data from English-language randomized controlled trials (RCTs) to evaluate the efficacy and safety of omalizumab in patients aged 6–20 years with allergic asthma, with specific attention to age-stratified outcomes where feasible.

**Methods:**

Conducted in accordance with the PRISMA 2020 guidelines, this review followed a pre-registered protocol (PROSPERO: CRD420251132811). Eligible studies were English-language RCTs involving predominantly pediatric and adolescent patients with allergic asthma; one included trial enrolled participants up to 20 years of age, but only 15 participants (0.8% of the total sample) were aged 18–20 years, and all remaining participants were <18 years. Studies compared omalizumab with control treatment (placebo or standard therapy). Efficacy outcomes included asthma exacerbations, forced expiratory volume in 1 s (FEV1), and Childhood Asthma Control Test (C-ACT) scores. Safety outcomes encompassed treatment-emergent adverse events (TEAEs), serious adverse events (SAEs), and asthma-related hospitalizations. Data were pooled using random-effects models where appropriate, with heterogeneity assessed via I^2^ statistics.

**Results:**

Six RCTs involving 1,835 participants (omalizumab: 1,134; control: 701) aged 6–20 years were included. Omalizumab significantly reduced asthma exacerbations (relative risk RR = 0.66, 95% CI: 0.55-0.79; I^2^ = 51.6%), SAEs (RR = 0.56, 95% CI: 0.35–0.90; I^2^ = 43.1%), and hospitalizations (RR = 0.35, 95% CI: 0.22–0.56; I^2^ = 0.0%). No significant difference was found in TEAEs (RR = 0.96, 95% CI: 0.92–1.01; I^2^ = 1.5%). Evidence regarding lung function and asthma control was limited and could not be quantitatively pooled.

**Conclusion:**

Omalizumab is effective in reducing exacerbations, SAEs, and hospitalizations in pediatric and adolescent allergic asthma, with a favorable safety profile. Evidence regarding lung function and asthma control remains limited and should be interpreted cautiously. These findings support its use as an add-on therapy for moderate-to-severe disease inadequately controlled by standard treatments.

**Systematic Review Registration:**

https://www.crd.york.ac.uk/PROSPERO/view/CRD420251132811, identifer CRD420251132811.

## 1 Introduction

Pediatric and adolescent allergic asthma is a pressing global public health challenge, affecting approximately 10%–15% of individuals under 18 years of age (Asher et al., 2006). The condition is associated with recurrent airflow limitation, bronchial hyper-responsiveness, and chronic airway inflammation, leading to missed school days, reduced quality of life, and increased healthcare utilization (O'Connell, 2004; Yamasaki et al., 2020). Although standard therapies—including ICS, LABA, and leukotriene receptor antagonists—form the cornerstone of management, 20%–30% of patients with moderate-to-severe persistent allergic asthma fail to achieve adequate disease control, highlighting a significant unmet therapeutic need (Humbert et al., 2005).

Omalizumab, a humanized monoclonal antibody that binds free IgE, was introduced in the early 2000s as a targeted biologic for moderate-to-severe allergic asthma (Shen et al., 2025). By inhibiting IgE binding to high-affinity receptors (FcεRI) on mast cells and basophils, omalizumab reduces allergen-induced degranulation, inflammatory cytokine release, and airway remodeling—mechanisms particularly relevant in pediatric populations where IgE-mediated inflammation drives disease pathogenesis (Serrano-Candelas et al., 2015; Galli et al., 2025). Numerous RCTs have evaluated omalizumab in children and adolescents, but reported efficacy has varied, possibly due to differences in study design, patient characteristics, and outcome definitions (Núria et al., 2013). These inconsistencies underscore the need for a rigorous synthesis of evidence.

Previous meta-analyses on omalizumab in pediatric asthma have notable limitations, including the inclusion of non-English studies—which may introduce translation bias—and insufficient age-stratified reporting, thereby obscuring potential differences between children and adolescents (Cheng et al., 2023; Li et al., 2023; Li et al., 2024; Liao et al., 2024). Furthermore, few reviews have focused exclusively on English-language RCTs, which are widely accessed in international clinical decision-making due to standardized reporting (Liberati et al., 2009).

To address these gaps, this systematic review and meta-analysis synthesizes evidence from English-language RCTs on the efficacy and safety of omalizumab in pediatric and adolescent allergic asthma, with explicit attention to age subgroups where data permit. The findings aim to inform clinical guidelines and support personalized treatment strategies.

## 2 Methods

### 2.1 Protocol registration and reporting guidelines

This systematic review and meta-analysis was conducted in strict compliance with the Preferred Reporting Items for Systematic Reviews and Meta-Analyses (PRISMA) 2020 guidelines (Moher et al., 2009). The study protocol was prospectively registered in the International Prospective Register of Systematic Reviews (PROSPERO) under the registration number CRD420251132811.

### 2.2 Eligibility criteria

Studies were selected based on the PICOS framework.

- Population: Pediatric/adolescent patients (≤20 years) with physician-diagnosed allergic asthma, confirmed by skin prick test, elevated serum IgE, or clinical history. Studies including non-allergic asthma or patients >20 years were excluded.
- Intervention: Omalizumab administered subcutaneously at any approved dose or interval.
- Comparison: Placebo (with or without standard therapy) or active standard therapy (e.g., budesonide/formoterol).
- Outcomes: Efficacy: asthma exacerbations, FEV1, % predicted, C-ACT scores. Safety: TEAEs, SAEs, asthma-related hospitalizations.
- Study design: English-language RCTs (parallel-group preferred). Non-randomized, observational, and non-English studies were excluded.

### 2.3 Search strategy

A systematic search was performed in PubMed, Embase, Cochrane CENTRAL, and Web of Science without date restrictions, limited to English. Search terms included “pediatric asthma,” “adolescent asthma,” “omalizumab,” “anti-IgE,” and “randomized controlled trial.” Reference lists of relevant reviews and clinical trial registries (ClinicalTrials.gov, EU Clinical Trials Register) were also scanned. The detailed search strategy for Pubmed database is provided in the Supplementary Material (Supplementary Material).

### 2.4 Study selection and data extraction

Two reviewers independently screened titles/abstracts and full texts. Discrepancies were resolved by consensus or third-reviewer consultation. A standardized form was used to extract study characteristics, participant data, and outcome measures.

### 2.5 Risk of bias assessment

The Cochrane Risk of Bias tool (Higgins et al., 2011) was applied independently by two reviewers across seven domains: random sequence generation, allocation concealment, blinding of participants and personnel, blinding of outcome assessment, incomplete outcome data, selective reporting, and other bias.

### 2.6 Statistical analysis

Analyses were performed using Stata 18.0. Dichotomous outcomes were pooled as RRs with 95% CIs; continuous outcomes as SMDs. Heterogeneity was assessed via I^2^, with values >40% considered indicative of moderate heterogeneity (Migliavaca et al., 2022). Random-effects models were used throughout. Sensitivity analyses involved sequentially excluding each study. Pre-specified subgroup analyses by age (<12 vs. ≥12 years), baseline IgE level, and treatment duration could not be performed due to insufficient data (e.g., only one study included adolescents exclusively, and IgE thresholds varied across studies). These analyses are therefore not reported. Statistical significance was set at p < 0.05.

## 3 Results

### 3.1 Study selection and characteristics

The literature search and screening process, conducted in accordance with PRISMA 2020 guidelines, identified six English-language RCTs (Milgrom et al., 2001; Lanier et al., 2009; Massanari et al., 2009; Kulus et al., 2010; Busse et al., 2011; Chen et al., 2023) that met all eligibility criteria for inclusion in the meta-analysis. The flowchart shows the process of study selection (Figure 1). Studies were published between 2001 and 2023 and conducted across multiple countries. Participants were aged 6–20 years with moderate-to-severe persistent allergic asthma; only 15 participants (0.8%) were aged 18–20 years in the Busse study, and all other participants were <18 years. Omalizumab dosing varied across studies, including subcutaneous fixed-dose regimens of 75–375 mg every 2–4 weeks, 75–375 mg every 1–2 months, and 150–600 mg as reported in the included trials, as well as individualized weight- and IgE-based regimens of at least 0.016 mg/kg per IU/mL IgE monthly or every 4 weeks. Control arms included placebo plus ICS/LABA or active comparator. Follow-up ranged from 16 to 60 weeks. Study characteristics are summarized in Table 1.

**FIGURE 1:**
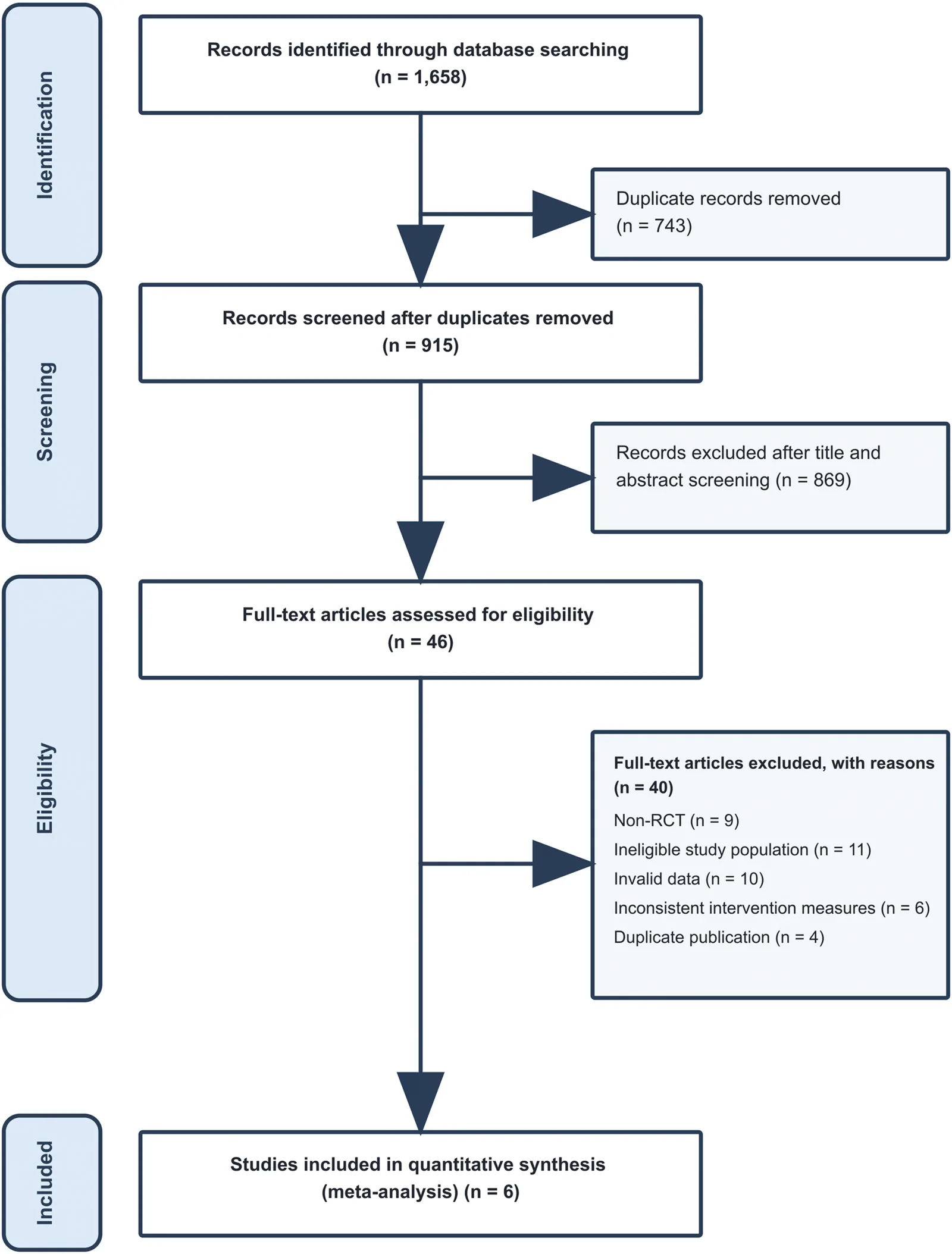
Flow chart of the literature search.

**TABLE 1:** Baseline characteristics and primary results of included trials.

Author, year	Location	Sample	Age (year)	Asthma severity	Interventions	Control	Duration (week)	Outcome
Busse et al. (2011)	USA	208/211	6-20	Allergic asthma	SC 75–375 mg q2–4wk	Placebo + basic treatment	60	①②③④⑤⑥
Chen et al. (2023)	China	44/44	6-11	Moderate to severe	SC 150–600 mg qd	Budesonide/formoterol inhalation therapy	16	②③④
Kulus et al. (2010)	Multiple countries	159/76	6-12	Severe persistent	SC 75–375 mg q1–2mo	Placebo + high-dose ICS + LABA	52	①④⑤⑥
Lanier et al. (2009)	USA, Poland	421/207	6-12	Moderate to severe persistent	SC 75–375 mg q2–4wk	Placebo + ICS	52	①④⑥
Massanari et al. (2009)	USA	76/70	12-17	Moderate to severe	SC (min. 0.016 mg/kg/(IU/mL IgE) monthly) + ICS	Placebo + baseline treatment	28	④⑤⑥
Milgrom et al. (2001)	USA	225/109	6-12	Moderate to severe	SC (min. 0.016 mg/kg/(IU/mL IgE) q4wk) + BDP	Placebo + BDP	28	①④⑤⑥

Abbreviations: BDP: beclomethasone dipropionate; ICS: inhaled corticosteroids; LABA: long-acting β₂ agonists; SC: subcutaneous injection. Outcome: ① = Exacerbation; ② = FEV1; ③ = C-CAT; ④ = TMAEs; ⑤ = SAEs; ⑥ = Hospitalization.

### 3.2 Risk of bias assessment

Using the Cochrane Risk of Bias tool, we assessed seven bias domains for included studies (summarized in Figures 2, 3). Most studies demonstrated low risk in blinding and outcome reporting. Some unclear risks were noted in randomization and allocation concealment due to insufficient methodological detail.

**FIGURE 2:**
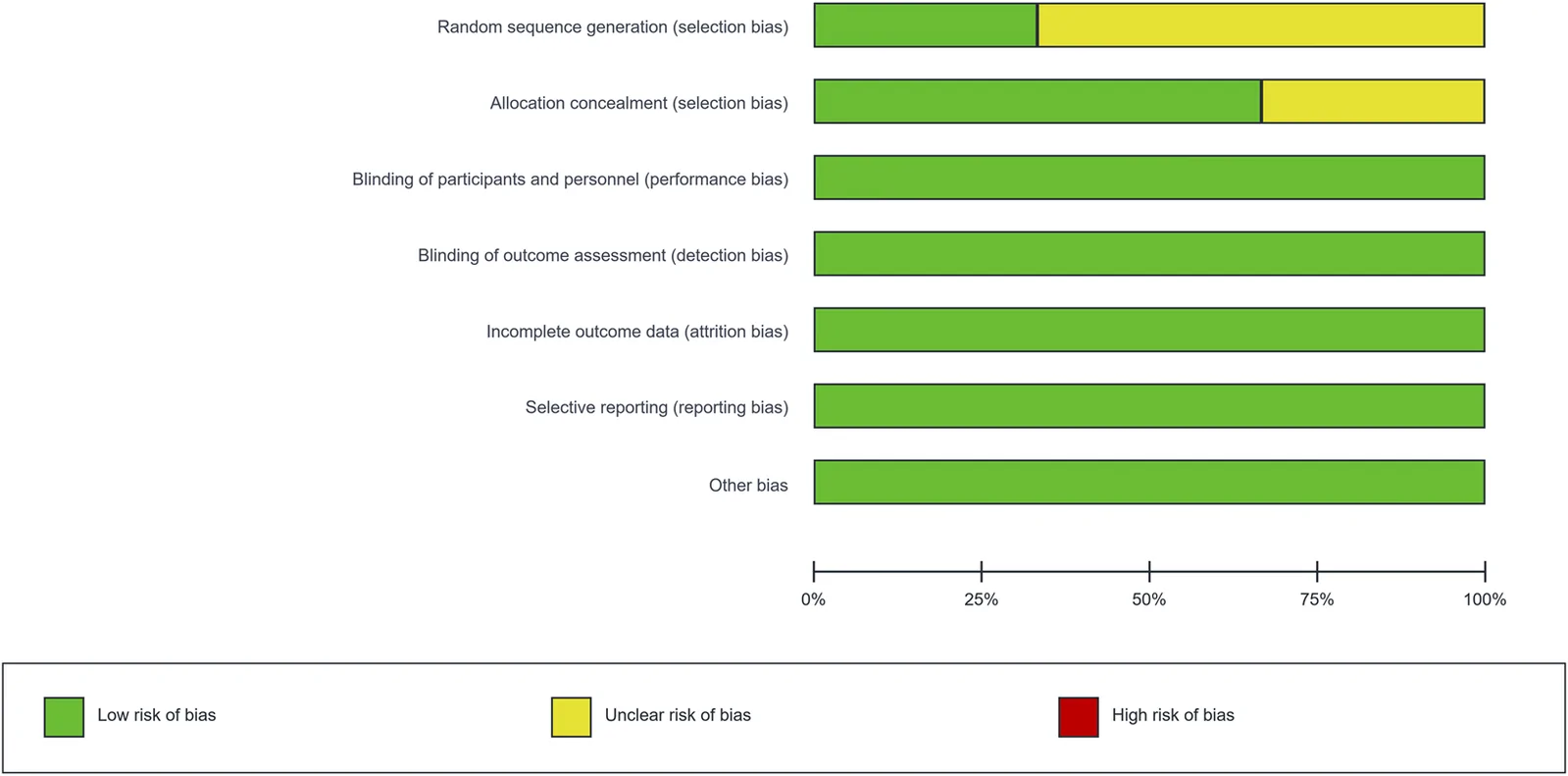
Risk of bias graph.

**FIGURE 3:**
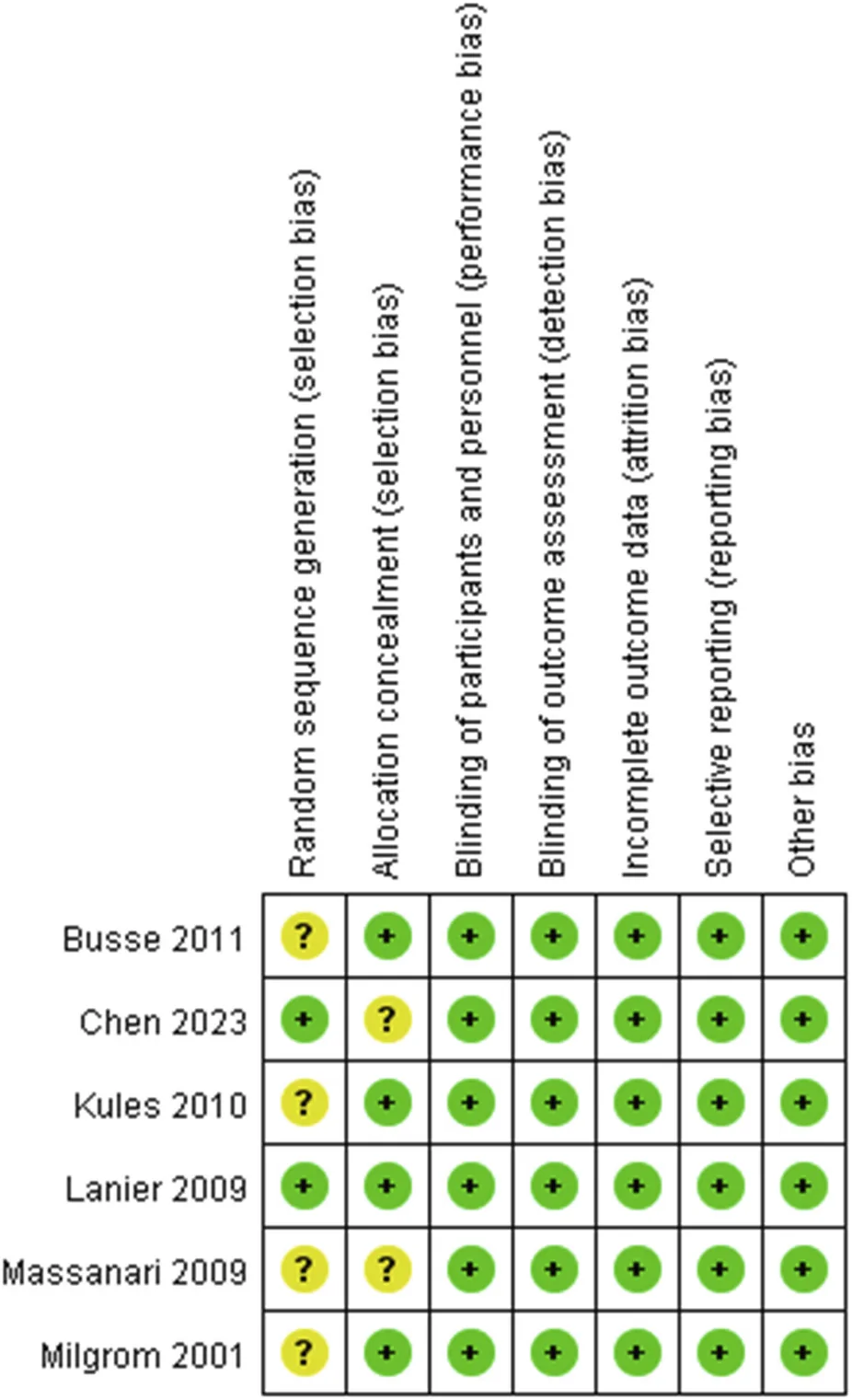
Risk of bias summary.

### 3.3 Efficacy outcomes

Four RCTs reported asthma exacerbations. Omalizumab significantly reduced the risk of exacerbations compared with control interventions (relative risk RR = 0.66, 95% CI: 0.55-0.79; I^2^ = 51.6%; Figure 4). For FEV1 and C-ACT scores, quantitative pooling was not performed because only two studies reported these outcomes and substantial clinical and statistical heterogeneity was present, including differences in comparator type and outcome reporting. Therefore, these outcomes were summarized narratively. Overall, the available evidence suggested that omalizumab may improve lung function and asthma control; however, the certainty of these findings is limited by the small number of studies and heterogeneity across trials.

**FIGURE 4:**
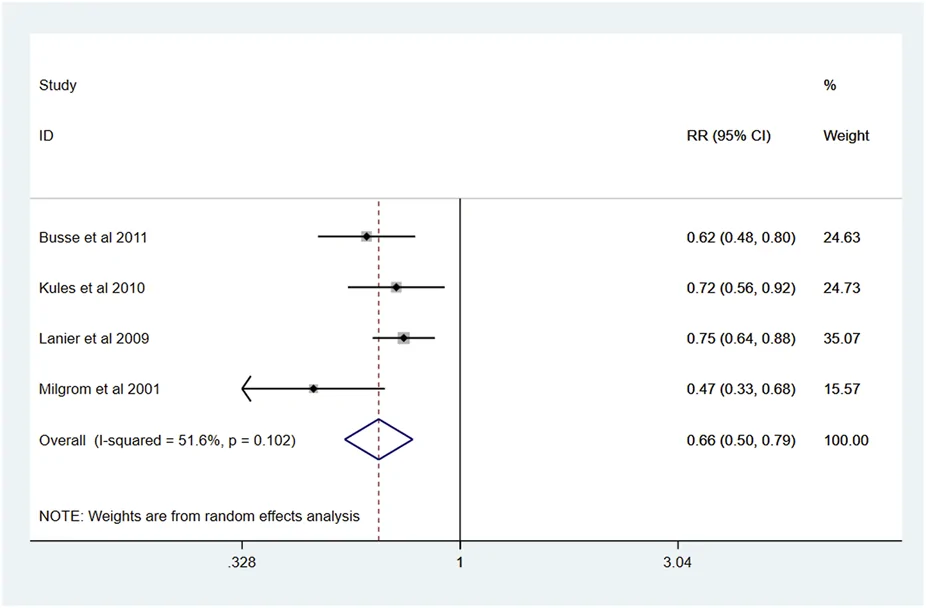
Forest plot of exacerbation.

### 3.4 Safety outcomes

No significant difference was found in TEAEs (RR = 0.96, 95% CI: 0.92–1.01; I^2^ = 1.5%; Figure 5). Omalizumab reduced SAEs (RR = 0.56, 95% CI: 0.35–0.90; I^2^ = 43.1%; Figure 6) and hospitalizations (RR = 0.35, 95% CI: 0.22–0.56; I^2^ = 0.0%; Figure 7).

**FIGURE 5:**
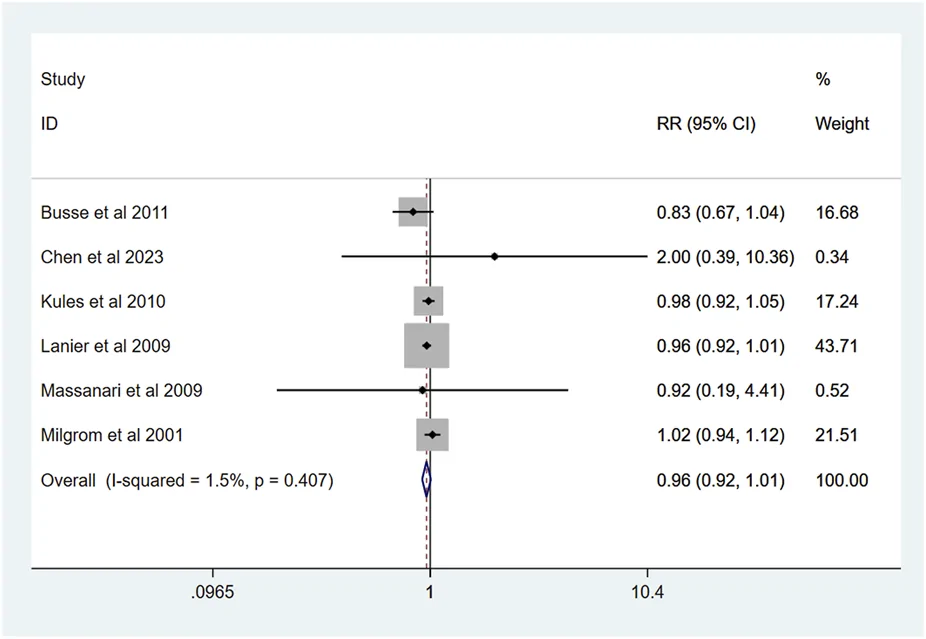
Forest plot of TEAEs.

**FIGURE 6:**
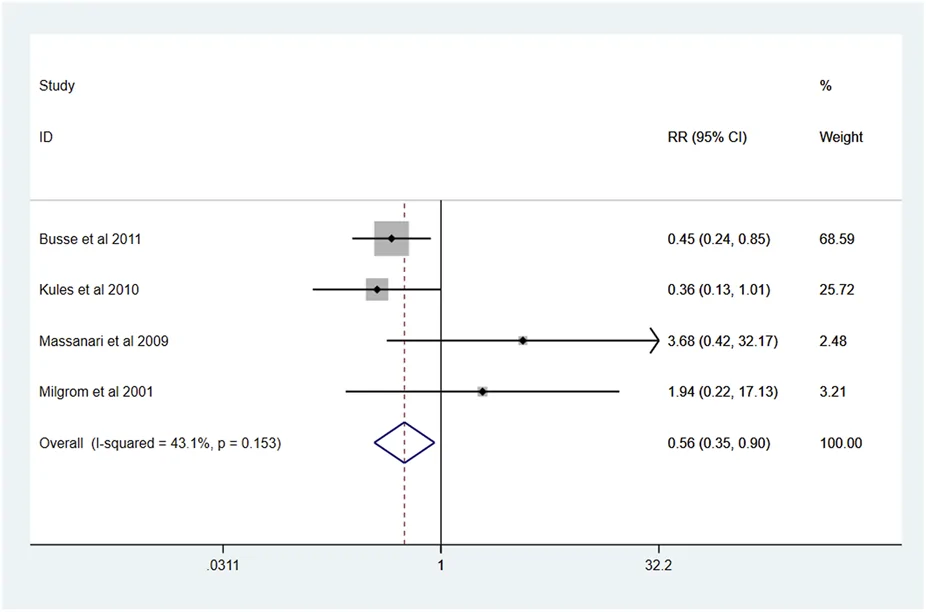
Forest plot of SAEs.

**FIGURE 7:**
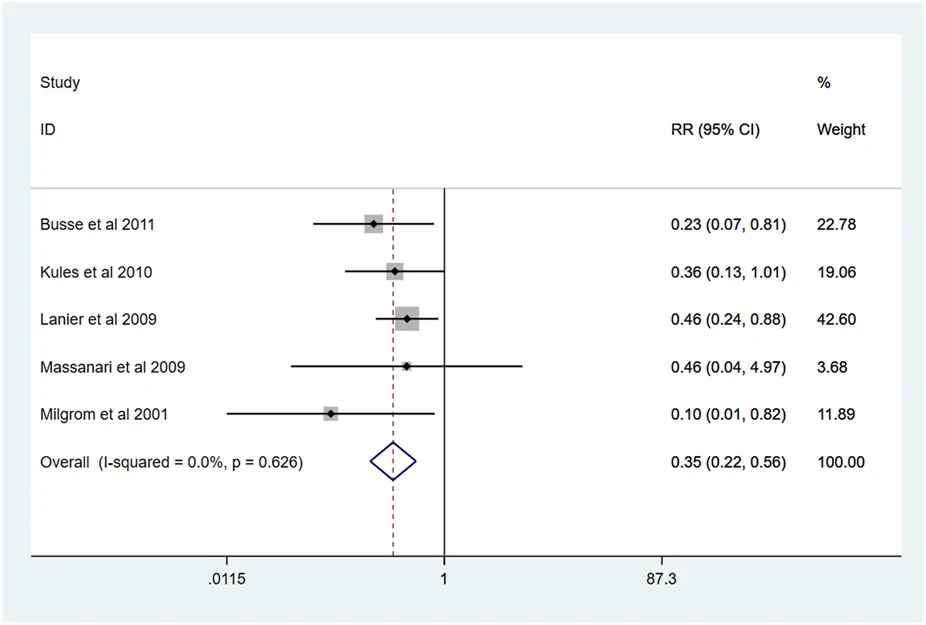
Forest plot of hospitalization.

### 3.5 Sensitivity analyses

Sensitivity analyses, performed by iteratively excluding each study, confirmed the overall direction of effect for asthma exacerbations, with recalculated RRs ranging from 0.49 (95% CI: 0.36–0.67) to 0.84 (95% CI: 0.69–1.02) and the effect direction remaining unchanged (Supplementary Figure S1). However, the upper confidence limit crossed the null when Lanier et al., 2009 was excluded, suggesting that the estimate should be interpreted with some caution given the limited number of studies. Similarly, for hospitalization, the RRs varied between 0.13 (95% CI: 0.04–0.41) and 0.63 (95% CI: 0.38–1.04; Supplementary Figure S2), consistently favoring omalizumab. Analyses of TEAEs (Supplementary Figure S3), and SAEs (Supplementary Figure S4) also indicated no influential outliers.

### 3.6 Publication bias

For the exacerbation outcome (four studies), visual inspection of the funnel plot (Supplementary Figure S5) showed some asymmetry. Egger’s test (p = 0.097) and Begg’s test (p = 0.089) were not statistically significant. However, given the small number of studies, these tests have low power, and no firm conclusion on publication bias can be drawn.

## 4 Discussion

The present meta-analysis synthesizes evidence from six English-language RCTs to evaluate omalizumab?s efficacy and safety in pediatric and adolescent patients with allergic asthma-a population where standard therapies often fail to achieve adequate control. Our findings demonstrate that omalizumab significantly reduces asthma exacerbations, SAEs, and hospitalizations without increasing TEAEs. Evidence regarding lung function (FEV1) and asthma control (C-ACT) was limited and was summarized narratively rather than pooled quantitatively. Overall, these findings support omalizumab?s role as a safe and effective add-on therapeutic option for this vulnerable group, while indicating that lung function and asthma control outcomes require further confirmation.

Our pooled estimate for exacerbation reduction (RR 0.66; 95% CI: 0.55-0.79) aligns with previous meta-analyses, though with critical distinctions. Lang et al. (Li et al., 2023) included seven RCTs (including non-English studies) and reported a similar RR of 0.55 (95% CI: 0.35–0.85), but did not stratify by age—masking potential differences between children and adolescents. Only one study (Massanari et al., 2009) focused exclusively on adolescents (12–17 years), in that trial, no exacerbations were recorded in either arm, making it impossible to estimate a relative risk or perform a formal subgroup comparison with younger children. Despite this, adolescent asthma remains associated with poorer treatment adherence and higher exacerbation risk (Anderson et al., 2024), highlighting a potential role for omalizumab in this age group based on the broader evidence.

For lung function and asthma control outcomes, the available evidence was limited. Only two studies reported FEV1 and C-ACT scores, and these studies differed substantially in comparator type and outcome reporting. Therefore, we did not perform quantitative pooling or report pooled effect estimates for these outcomes. Although the individual studies generally suggested potential improvements with omalizumab, these findings should be interpreted cautiously and require confirmation in future well-designed trials using standardized outcome measures.

In terms of safety, our finding of no increased TEAEs risk (RR 0.96; 95% CI: 0.92–1.01) is consistent with the global safety profile of omalizumab, which has been shown to have a similar adverse event profile to placebo in pediatric populations (Fenu et al., 2023). The significant reduction in SAEs (RR 0.56; 95% CI: 0.35–0.90) and hospitalizations (RR 0.35; 95% CI: 0.22–0.56) is particularly impactful, as these outcomes are key drivers of healthcare costs and patient morbidity (Jacobs et al., 2022). Notably, the zero heterogeneity in hospitalization results (I^2^ = 0.0%) suggests a consistent protective effect of omalizumab across studies—even those with varying follow-up durations and control arms.

Omalizumab’s efficacy in our analysis can be attributed to its targeted mechanism of action: binding to free IgE and preventing FcεRI cross-linking on mast cells and basophils (Kaplan et al., 2017; Pelaia et al., 2018). In pediatric allergic asthma, IgE levels correlate with disease severity and exacerbation risk (Haselkorn et al., 2010; Aziz et al., 2023), making anti-IgE therapy a rational choice. Additionally, omalizumab reduces eosinophilic airway inflammation—a key feature of uncontrolled asthma—by inhibiting IgE-mediated recruitment of eosinophils (Busse et al., 2020; Yan et al., 2023). This mechanism may explain the observed improvements in FEV1 and C-ACT scores, as reduced inflammation translates to better airflow and symptom control.

Notably, two of our included studies used weight- and IgE-tailored doses of omalizumab (Milgrom et al., 2001; Massanari et al., 2009), which are now recommended in clinical guidelines to optimize efficacy (Menzella et al., 2023). While our meta-analysis did not formally compare fixed vs. tailored dosing, the consistent efficacy across studies suggests that both approaches may be effective—though future studies should explore whether tailored dosing improves outcomes in specific subgroups (e.g., low IgE, young children).

Our findings have important implications for clinical practice and guideline development. First, omalizumab’s ability to reduce exacerbations and hospitalizations supports its use in children and adolescents with moderate-to-severe allergic asthma who are uncontrolled on ICS/LABA—a population currently identified as a priority in GINA (Venkatesan, 2025). Second, because age-stratified analyses could not be performed, no conclusions about age-related differences in efficacy can be drawn. Additional age-stratified trials are needed. Third, the safety profile of omalizumab—particularly the absence of increased TEAEs—may alleviate concerns among clinicians and caregivers about biologic therapy in pediatric populations. While anaphylaxis is a rare but serious adverse event associated with omalizumab, none of our included studies reported anaphylaxis cases, consistent with its low incidence (Cox et al., 2007). This, combined with the reduction in SAEs, positions omalizumab as a favorable option for long-term management.

Despite methodological rigor, our study has several limitations. First, only six RCTs were included, limiting statistical power for subgroup analyses (e.g., IgE levels, comorbid atopic dermatitis). This is partly due to our focus on English-language studies, which excluded potentially relevant non-English RCTs-though this choice minimized translation bias and improved generalizability to global clinical practice (Dobrescu et al., 2021; Konno et al., 2020). Second, FEV1 and C-ACT outcomes were reported by only two studies and showed substantial clinical and statistical heterogeneity; therefore, these outcomes were summarized narratively rather than pooled quantitatively. Third, most studies included children aged ≥ 6 years, with no data on children <6 years-a population with unmet needs, as ICS adherence is particularly challenging in toddlers (Vasbinder et al., 2016; Bach et al., 2022); only one study focused exclusively on adolescents. This limits generalizability to adolescents and younger children. Fourth, follow-up durations varied from 16 to 60 weeks, with no long-term (≥ 1 year) data on omalizumab?s efficacy or safety-critical for assessing sustained benefits and potential late adverse events (e.g., immunogenicity). Fifth, although one study (Busse et al., 2011) enrolled participants up to 20 years of age, only 15 individuals (0.8%) were aged 18–20 years; this small number does not materially affect the pediatric focus of our findings, but it is a deviation from the strict <18 years criterion and is noted as a limitation. Sixth, the included studies used either fixed doses (75–375 mg) or weight/IgE-based dosing. Both appeared effective, but current guidelines favour individualized dosing based on IgE and body weight. Without head-to-head comparisons, optimal dosing remains uncertain. Future research should examine whether tailored dosing benefits specific subgroups, including children with low IgE or younger age. Seventh, our restriction to English-language RCTs may limit generalizability to non-Western pediatric populations, where disease prevalence, allergen profiles, and healthcare systems differ. However, this approach minimized translation bias and ensured consistency in outcome reporting. Finally, some studies had unclear risk of bias for allocation concealment or blinding, which may have introduced subtle biases in outcome reporting.

Future studies should address these limitations by: (1) conducting large, multicenter RCTs of omalizumab in children <6 years to fill the age gap in current evidence; (2) evaluating long-term (≥2 years) efficacy and safety to inform sustained treatment decisions; (3) exploring subgroup effects of baseline IgE levels, comorbid allergies, and adherence to identify patients most likely to benefit from omalizumab; and (4) including non-English studies in future meta-analyses (with rigorous translation and quality assessment) to expand generalizability. Additionally, real-world studies are needed to complement RCT data, as they better reflect routine clinical practice (e.g., diverse patient populations, comorbidities) and can assess long-term outcomes such as QoL and healthcare costs.

## 5 Conclusion

This meta-analysis supports omalizumab as an effective and safe add-on therapy for moderate-to-severe pediatric and adolescent allergic asthma inadequately controlled by standard treatments. The results provide evidence for clinical guideline updates and personalized management approaches.

## Data Availability

The original contributions presented in the study are included in the article/Supplementary Material, further inquiries can be directed to the corresponding author.
