# Supplementary material for: Omalizumab for pediatric allergic asthma: evidence-based insights on efficacy and safety from a meta-analysis of randomized trials
Source: Front Pharmacol. 2026 Sep 24;17:1795193. doi: 10.3389/fphar.2026.1795193 (PMC13646124; doi:10.3389/fphar.2026.1795193)
Supplement: Supplementary file 1 [file Table1.docx]

### ****Supplementary material****

****Supplementary methods Full Electronic Search Strategy of Pubmed****

#1	"Asthma"[Mesh] OR "Asthma, Allergic"[Mesh] OR "Pediatric Asthma"[Mesh] OR "Childhood Asthma"[Mesh]

#2	"Asthma"[tiab] OR "allergic asthma"[tiab] OR "atopic asthma"[tiab] OR "pediatric asthma"[tiab] OR "childhood asthma"[tiab] OR "adolescent asthma"[tiab] OR "paediatric asthma"[tiab]

#3	#3 #1 OR #2

#4	"Omalizumab"[Mesh] OR "Anti-IgE Antibody"[Mesh]

#5	"omalizumab"[tiab] OR "Xolair"[tiab] OR "anti-IgE"[tiab] OR "anti IgE"[tiab] OR "immunoglobulin E inhibitor*"[tiab]

#6	#4 OR #5

#7	"Randomized Controlled Trial"[Publication Type] OR "Controlled Clinical Trial"[Publication Type] OR "randomized"[tiab] OR "randomised"[tiab] OR "placebo"[tiab] OR "randomly"[tiab] OR "trial"[tiab] OR "RCT"[tiab] OR "groups"[tiab]

#8	"child"[tiab] OR "children"[tiab] OR "pediatric"[tiab] OR "paediatric"[tiab] OR "adolescent"[tiab] OR "teen"[tiab] OR "youth"[tiab] OR "boy"[tiab] OR "girl"[tiab] OR "school-age"[tiab]

#9	#3 AND #6 AND #7 AND #8

#10 Filters: English
